# Kiwifruit in the Omics Age: Advances in Genomics, Breeding, and Beyond

**DOI:** 10.3390/plants13152156

**Published:** 2024-08-03

**Authors:** Mian Faisal Nazir, Jinpeng Lou, Yu Wang, Shuaiyu Zou, Hongwen Huang

**Affiliations:** 1 Key Laboratory of Ex Situ Plant Conservation and Utilization of Jiangxi Province, Lushan Botanical Garden, Chinese Academy of Sciences, Jiujiang 330022, China; mfn121@hotmail.com (M.F.N.);; 2 South China Botanical Garden, Chinese Academy of Sciences, Guangzhou 510650, China; 3 University of Chinese Academy of Sciences, Beijing 100049, China

**Keywords:** kiwifruit, breeding, multi-omics, genetic diversity, trait improvement

## Abstract

The kiwifruit, *Actinidia* genus, has emerged as a nutritionally rich and economically significant crop with a history rooted in China. This review paper examines the global journey of the kiwifruit, its genetic diversity, and the role of advanced breeding techniques in its cultivation and improvement. The expansion of kiwifruit cultivation from China to New Zealand, Italy, Chile and beyond, driven by the development of new cultivars and improved agricultural practices, is discussed, highlighting the fruit’s high content of vitamins C, E, and K. The genetic resources within the *Actinidia* genus are reviewed, with emphasis on the potential of this diversity in breeding programs. The review provides extensive coverage to the application of modern omics technologies, including genomics, transcriptomics, proteomics, and metabolomics, which have revolutionized the understanding of the biology of kiwifruit and facilitated targeted breeding efforts. It examines both conventional breeding methods and modern approaches, like marker-assisted selection, genomic selection, mutation breeding, and the potential of CRISPR-Cas9 technology for precise trait enhancement. Special attention is paid to interspecific hybridization and cisgenesis as strategies for incorporating beneficial traits and developing superior kiwifruit varieties. This comprehensive synthesis not only sheds light on the current state of kiwifruit research and breeding, but also outlines the future directions and challenges in the field, underscoring the importance of integrating traditional and omics-based approaches to meet the demands of a changing global climate and market preferences.

## 1. Introduction

Kiwifruits belong to the genus *Actinidia* in the family Actinidiaceae [1]. They originated in the Yangtze River valley and surrounding mountain ranges in central and eastern China [2]. Around 55 species of *Actinidia* have been identified, but only two are widely grown commercially—*A. deliciosa* and *A. chinensis* [3]. The ploidy level of *Actinidia* species varies, typically ranging from diploid (2x) to hexaploid (8x), which contributes to their genetic diversity and adaptability [4]. Ploidy manipulation, such as colchicine-induced polyploidy, is used to enhance traits like fruit size and disease resistance, while interspecific hybridization and molecular markers aid in managing and exploiting this genetic diversity [5]. Kiwifruits are perennial dioecious vines, with separate male and female plants [6]. The vines can grow over 10 m long and the leaves are oval in shape with pointed tips. Kiwifruit flowers are small, white, or yellow, and give rise to oval or cylindrical berries containing numerous small, black seeds surrounded by juicy, green, yellow, or red flesh and a thin, slightly fuzzy skin. The flesh has a unique sweet–tart flavor and aromatic qualities.

Kiwifruits have been consumed in China for centuries, where various species grow in the wild [7]. The first recorded cultivation of kiwifruit was in the 12th century during the Tang dynasty in China. Over the centuries, many wild *Actinidia* species were brought into cultivation in China [8]. In the early 20th century, seeds of *A. deliciosa* were transported from China to New Zealand. A horticulturist named Hayward Wright developed the cultivar ‘Hayward’, which became the foundation of the New Zealand kiwifruit industry starting in the 1970s [9]. Italy, Chile, France, Japan, and the United States also established commercial kiwifruit production over the late 20th century [10]. Global kiwifruit production now exceeds 3 million tons harvested from over 90,000 hectares [11]. The main producers are China, Italy, New Zealand, and Chile, but many other countries also grow kiwifruit commercially. Kiwifruits are nutritionally dense, providing significant amounts of vitamin C, vitamin E, vitamin K, folate, potassium, and dietary fiber [12,13,14,15]. The global kiwifruit market continues to grow steadily as production expands and new cultivars are developed.

Modern omics tools and technologies are rapidly advancing kiwifruit research and enabling more targeted breeding efforts [16]. Genomic resources have expanded dramatically, with high-quality reference genomes now available for key kiwifruit species and cultivars [17]. These genome sequences provide insights into the genetic factors influencing important horticultural traits and underpin marker-assisted breeding. Transcriptomics analyses using RNA-seq have been widely applied to identify genes and pathways involved in fruit development, ripening, aroma/flavor biosynthesis, phytochemical accumulation, and stress responses. Proteomic profiling is also being used to better understand kiwifruit protein composition and metabolism. Metabolomic analyses have revealed complex biochemical changes during fruit development and storage. Together, these system-wide omics datasets are unraveling the molecular basis of key traits.

Integrating multi-omics data through systems biology approaches allows the reconstruction of molecular networks regulating complex polygenic traits. This is strengthening functional gene discovery and marker-trait associations for genomic selection. High-throughput phenotyping technologies are enabling more rapid and accurate characterizations of diverse kiwifruit germplasm for breeding. Genome editing has emerged as a promising new tool for the targeted improvement of specific fruit traits. Furthermore, innovations in sterile tissue culture and cryopreservation are enhancing the maintenance, exchange, and utilization of valuable kiwifruit genetic resources.

This review synthesizes the current knowledge on the application of genomic, transcriptomic, proteomic, and metabolomic technologies to elucidate genetic factors controlling agronomic traits in kiwifruit. Key findings from omics-enabled research underpinning kiwifruit quality, phytochemical content, biotic/abiotic stress adaptation, yield, flavor, ripening behavior, and storage potential are discussed. This review examines how modern omics strategies are improving germplasm evaluation, genetic diversity characterization, gene discovery, molecular marker development, genotype–phenotype associations, and genomic selection accuracy of kiwifruit. The major challenges and future outlook for omics-accelerated breeding in kiwifruit are also considered. Overall, this review highlights the vital role that systems biology and omics tools are playing in unraveling kiwifruit biology and driving precision breeding for continued germplasm enhancement.

## 2. Kiwifruit Genetic Resources

China is recognized as the center of origin and diversity for the genus *Actinidia*. Around 55 species have been identified, most native to central, southern, and western China [3,18]. Most of the reported species available in China are presented in Table 1, along with their feature characteristics. Additional *Actinidia* species occur in adjacent regions, including northern Myanmar, Vietnam, and eastern Himalaya [19,20]. Significant genetic and phenotypic diversities exist within *Actinidia*. They show wide variation in characteristics like plant morphology, flowering time, fruit types, flavor profiles, and environmental adaptation [21,22,23,24]. A few key commercially relevant species include:

*A. chinensis*—Chinese gooseberry [1]. Small, round fruit with smooth, thin skin and a very sweet taste. Important female parent for yellow-fleshed ‘gold’ kiwifruit. Originated in central China.

*A. deliciosa*—Hardy kiwifruit [25]. Large, oval fruit with fuzzy skin and a more tangy taste. Principal green-fleshed variety grown globally. Native to southeastern China.

*A. arguta*—Tender kiwifruit [26,27]. Oblong smooth-skinned fruit. Tropical/subtropical regions.

*A. kolomikta*—Arctic kiwifruit [28]. Tolerates colder climates. Introduced red-flesh trait to breeding.

Many other wild species offer potential for use in breeding as well, such as *A. indochinensis*, *A. polygama*, *A. glaucophylla*, *A. melanandra*, and *A. eriantha* [29,30]. These wild relatives possess genetic diversity for a range of valuable horticultural traits and disease resistances that can be introgressed into new kiwifruit cultivars.

Germplasm collections help preserve *Actinidia* genetic resources and provide material for kiwifruit improvement. Major kiwifruit germplasm collections exist in China, Italy, New Zealand, Japan, Korea, and Chile [10]. These contain diverse *Actinidia* accessions collected from wild populations as well as released cultivars. For example, the Wuhan National Germplasm Repository in China holds over 1300 accessions from 28 *Actinidia* species [31,32]. Molecular marker analysis demonstrates these collections capture substantial *Actinidia* genetic diversity [33,34,35,36,37]. Trait evaluations also assess variations in characteristics like fruit quality, yield, flavor, and disease resistance [38,39,40]. Core collections have been established with representative diversity for efficient utilization in breeding. In addition to ex situ germplasm collections, in situ conservation efforts maintain *Actinidia* species within native forest ecosystems [41,41,42]. These complementary conservation strategies aim to preserve the full range of existing genetic diversity in *Actinidia*.

Overall, *Actinidia* species represent a rich source of genetic variation for kiwifruit breeding. Extensive germplasm collections serve as repositories of this diversity. The ongoing collection, characterization, and evaluation of wild *Actinidia* resources along with diverse kiwifruit cultivars will provide the genetic foundation for continued improvements. The introgression of desirable genes from wild species will enable the development of new kiwifruit cultivars with commercially valuable traits.

**Table 1:** Kiwifruit germplasm in China.

No.	Species (Genotype)	Distribution	Ploidy	Male/Female	Wild/Cultivated	Flesh Color	Features
1	*A. callosa*	Guangxi, China Wuhan, China	2x, 4x	Female	Cultivated	Jade green	Fruit size varies from medium to large with a very delicate and slightly acidic flavor
2	*A. diversicolora*	Sichuan, China	2x	Female	Wild		Variable leaf color. Found in temperate forests. Small, tart fruits used in local culinary dishes
3	*A. jiangxiensis*	Jiangxi, China	2x	Female	Wild	Green	
4	*A. arguta*	Guangxi, China Heilongjiang, China	4x, 8x	Female	-	Green	Smooth-skinned, apple-like fruit. High genetic variability, used in interspecific breeding
5	*A. pentapetala*	Guangxi, China	2x	Female	-		Small, sweet fruits with a unique penta-petalous flower structure. Grows in forested areas
6	*A. macrosperma*	Zhejiang, China	4x	Female	Wild	Orange	Oval fruits with relatively thicker skin and large seeds
7	*A. guilinensis*	Guangxi, China	2x	Female	-	Green	Produces medium-sized fruits with smooth skin. Known for its rich flavor and high nutritional content
8	*A. latifolia*	Shanxi, China Hubei, China Sichuan, China Guangxi, China	2x	Female	Cultivated	Jade green	High vitamin C content. Cultivated for its nutritional value and adaptability
9	*A. carnosifolia*	Guangxi, China		Male	Wild		
10	*A. hemsleyana*	Guangxi, China	2x	Female	Wild	Yellow green	Cylindrical fruits with smooth skin
11	*A. tetramera*	Guangdong, China	4x	Female	Wild		Small fruit. Adapted to mountainous regions. Known for its distinct flavor and smooth skin
12	*A. Eriantha*	Jiangxi, China Wuhan, China Guangxi, China	2x	Female	Cultivated	Jade green	High vitamin C content; easy peeling. Used for its smooth skin and nutritional benefits
13	*A. valvata*	Hunan, China	4x	Female	Wild		
14	*A. cylindrica*	Guangxi, China	2x	Female	-	Light green	Produces cylindrical fruits. Known for its unique shape and taste. Evergreen species
15	*A. polygamya*	Yunnan, China	4x	Female	Cultivated		
16	*A. indochinensis*	Guangxi, China	2x	Female	Cultivated	Green	Sub-globose fruit with smooth skin
17	*A. melliana*	Guangxi, China	2x	Female	Cultivated	Green	
18	*A. persicina*	Guangxi, China	2x	Female	-	Green	Produces peach-like fruits. Known for its unique flavor. Cultivated in various regions
19	*A. longicarpa*	Sichuan, China	2x	Female	-		Produces long fruits. Known for its unique shape and taste
20	*A. rongshuiensis*	Guangxi, China	2x	Female	-		Fruits are cylindrical and tomentose
21	*A. wantianensis*	Guangxi, China	2x	Female	-		Fruits are typically small and ovoid, with smooth skin
22	*A. chrysantha*	Guangxi, China	4x	Female	Wild	Green	Oval-shaped fruits are maroon–brown to greenish-brown
23	*A. rubricaulis*	Guangxi, China Sichuan, China	2x	Female	Cultivated		Small, oval fruits with yellow–red flesh color
24	*A. glaucophylla*	Guangxi, China	2x	Female	Wild	Green	Evergreen species with glaucous leaves
25	*A. liangguangensis*	Guangxi, China	2x	Female	Cultivated	Dark green	
26	*A. chinensis × A. eriantha*	Guangxi, China		Female	Cultivated	Green	
27	*A. albicalyx*	Guangxi, China	2x	Female	Wild		Evergreen species with unique foliage
28	*A. styracifolia*	Fujian, China	2x	Female	Cultivated		Berry-like fruits with smooth skin
29	*A. deliciosa*	Southeastern China	6x	Female	Cultivated	Green	Large, oval fruit with fuzzy skin and more tangy taste. Green-fleshed variety grown globally

The data in the table are sourced from Huang et al. [43], Qi et al. [4], and Wang et al. [44]. Ploidy levels: 2x = 58, 4x = 116, and 6x = 174, 8x = 232, where ‘x’ is the basic chromosome number (29) of the *Actinidia* species.

## 3. Modern Omics Approaches

A range of modern omics technologies have been applied to elucidate genetics, physiology, and metabolism in kiwifruit, as well as enable genomics-assisted breeding approaches.

### 3.1. Genomics

Molecular markers, including microsatellites (SSRs) and SNPs, have been widely used to assess genetic diversity among *Actinidia* species and cultivars [18,45,46,47]. High-density linkage maps have been constructed using these markers. For example, a linkage map of the crossbred *A. chinensis* var. chinensis ‘Hongyang’ × *A. chinensis* var. deliciosa ‘Qingyuanzhenzhu’ contained over 3000 SNP markers spanning 29 linkage groups [48]. Linkage maps have enabled QTL mapping studies to identify genomic regions associated with horticulturally important traits. Dozens of QTLs related to characteristics like fruit shape, soluble solids content, flesh color, ripening behavior, vitamin C levels, and Fusarium resistance have been mapped [48,49,50,51,52,53]. This sets the stage for marker-assisted breeding as well as identifying candidate genes’ underlying traits.

The Kiwifruit Genome Database (KGD) created by Junyang [54] is a comprehensive resource that includes publicly available genome and gene sequences, gene annotations, biochemical pathways, transcriptome profiles, and comparative genomic analysis results, facilitating extensive research in kiwifruit genomics. Another critical study by Zhang et al. identified sex-specific markers and narrowed down the sex-determination region (SDR) to a 1 Mb sub-telomeric region on chromosome 25, aiding in the accurate sex typing of male and female plants in breeding programs [49]. Yue et al. (2023) explored the origin and evolution of the kiwifruit Y chromosome, identifying sex-determining genes SyGl and FrBy and highlighting their roles in Y chromosome evolution through whole-genome comparisons [55]. Moreover, Akagi et al. investigated the recurrent neo-sex chromosome evolution in kiwifruit, identifying the key role of the *Shy Girl* gene and the impact of transposable elements in sex chromosome evolution [56].

Recent advancements in genomic resources for *Actinidia* species have paved the way for novel insights into their genetic makeup and evolutionary history (Table 2). High-quality genome assemblies for *A. chinensis* ‘Red5’ [57] and *A. deliciosa* ‘Hayward’ [58] have expanded upon the initial draft genome assemblies of *A. chinensis* ‘Hongyang’ [59,60] and *A. eriantha* ‘916’ [61], providing a deeper understanding of their genomic structures and gene content. These resources are crucial for the resequencing of diverse cultivars, enabling the detection of genetic variants and the discovery of molecular markers that facilitate targeted breeding programs.

**Table 2:** Overview of kiwifruit genomics studies.

Authors	Year	Key Findings
Crowhurst et al. [45]	2008	Identification of genes involved in flavor, health, color, and ripening through a cross-species EST database. This facilitated the understanding of genetic control over these traits and allowed for targeted breeding strategies.
Huang et al. [18]	2014	Demonstrated the benefits of natural hybridization and introgression in enhancing cultivar traits such as disease resistance, fruit quality, and yield.
Zhang et al. [49]	2015	Demonstrated the benefits of natural hybridization and introgression in enhancing cultivar traits such as disease resistance, fruit quality, and yield.
Wu et al. [60]	2019	Presented a high-quality genome sequence *of A. chinensis*, enhancing the precision of genetic studies and breeding programs.
Tahir et al. [51]	2020	Identification of QTLs linked to resistance against *Pseudomonas syringae* pv. *Actinidiae* (Psa), aiding in the development of canker-resistant kiwifruit varieties.
Yue et al. [54]	2020	Establishment of the Kiwifruit Genome Database, a resource that consolidates genomic data to support research and breeding programs.
Popowski et al. [48]	2021	Creation of a high-density genetic map, enabling the identification of QTLs for important traits such as fruit size and resistance to diseases, which is crucial for marker-assisted selection.
Lu et al. [46]	2022	Identification of a single nucleotide mutation controlling fruit flesh color, aiding in association mapping and breeding for desirable fruit traits.
Yao et al. [61]	2022	Conducted genome sequencing and comparative analysis of *A. eriantha*, enriching the genetic pool for breeding programs.
Li et al. [50]	2023	Development of a high-density genetic map and identification of QTLs associated with growth traits, facilitating the breeding of kiwifruit with optimized growth characteristics.
Wang et al. [52]	2023	Development of a comprehensive SNP genotyping array, enabling detailed genetic mapping and QTL analysis for traits like fruit quality and yield.
Akagi et al. [56]	2023	Discovery of recurrent neo-sex chromosome evolution, providing insights into the genetic mechanisms of sex chromosome development in kiwifruit.
Xia et al. [58]	2023	Achieved a chromosome-scale genome assembly, providing a high-resolution genetic resource for breeding and research.
Yue et al. [55]	2024	Detailed study on the origin and evolution of the Y chromosome in kiwifruit, enhancing the understanding of sex determination mechanisms.

The genome sequencing of different *Actinidia* species has been a pivotal step in the elucidation of genetic factors contributing to the diverse phenotypic traits observed in kiwifruit cultivars. The sequencing of the *A. chinensis* ‘*Hongyang*’ genome [59] resulted in a 653.86 Mb genome with 40,464 annotated protein-coding genes. This genome assembly has provided insights into the complex evolutionary history of the kiwifruit, including evidence of two whole-genome duplication events and a substantial portion of repetitive sequences, predominantly long terminal repeats, which underline the genetic diversity within the *Actinidiaceae* family.

In parallel, the sequencing of the *A. eriantha* ‘916’ genome has shed light on genome evolution and the structural variations between species [61]. With 41.3% of the genome consisting of repetitive elements, the study of *A. eriantha* has uncovered key differences in genes related to ascorbic acid biosynthesis and disease resistance, providing valuable information for breeding programs aimed at improving these traits in cultivated kiwifruit.

The *A. chinensis* ‘Red5’ cultivar, known for its distinctive red-fleshed fruit, has also had its genome sequenced, covering approximately 73% of the estimated genome size and significantly enhancing the quality of gene models over previous drafts [57]. This reference genome assists in the precise identification of quantitative trait loci (QTLs) and polymorphisms associated with important agronomic traits.

Comparative genomics among different cultivars and species of kiwifruit has uncovered structural variations and shed light on the genetic basis for the vast diversity of traits observed within the genus. The identification of expanded gene families involved in key biosynthetic pathways underscores the contributions of polyploidy to the enrichment of nutritional qualities in kiwifruit. With ongoing efforts to sequence more kiwifruit genomes, the breadth of genomic data will continue to grow, further enhancing the potential for scientific discovery and the improvement of kiwifruit cultivars through precision breeding. These expanding genomic resources are expected to catalyze a new wave of research, driving forward our comprehension of plant genetics, trait development, and evolution within *Actinidia* and related taxa.

### 3.2. Transcriptomics

Recent transcriptomic studies leveraging RNA-seq and microarray technologies have significantly enriched our understanding of the genetic underpinnings of kiwifruit development, ripening, and stress responses [62,63,64,65,66,67,68]. In-depth analyses of genome-wide gene expression in kiwifruit, such as those conducted on *A. chinensis* ‘Hongyang’, have identified over 6000 differentially expressed genes during various stages of fruit ripening [69,70,71]. These studies highlight the intricate regulation of genes involved in chlorophyll degradation [65,72], aroma volatile production [73,74], cell wall metabolism [66,68,75], and nutraceutical biosynthesis pathways [76,77], underscoring the dynamic changes in transcriptional activity from fruit development through ripening. Furthermore, the critical role of ethylene in kiwifruit ripening has been elucidated through the identification of key ethylene metabolism and signaling genes, including ACC synthase, ACC oxidase, and ethylene response factors, which exhibit regulated expression patterns coinciding with ripening stages [65]. The disassembly and modification of cell walls, a hallmark of fruit softening, have been attributed to the differential expression of genes encoding cell wall-modifying enzymes, revealing a complex regulation of cell wall metabolism during ripening [66]. Additionally, the interplay between various phytohormones, including cytokinins, auxins, and gibberellins, in kiwifruit development and ripening has been spotlighted, highlighting the hormonal cross-talk that finely tunes the ripening process [62]. Enrichment analysis and network construction from the transcriptomic data have further identified key metabolic pathways involved in sugar and vitamin C metabolism, critical for developing the fruit’s nutritional profile and taste. 

These transcriptomic insights not only advance our understanding of the molecular mechanisms governing kiwifruit development and ripening, but also provide valuable genetic markers and candidate genes for breeding programs aimed at improving fruit quality traits (Table 3). The integration of these findings offers a robust framework for future genetic and breeding efforts to enhance kiwifruit quality and stress resilience, paving the way for targeted genetic modifications to optimize desirable fruit attributes.

### 3.3. Proteomics

Recent advancements in proteomic techniques have provided invaluable insights into the protein profiles of kiwifruit tissues, illustrating the complex changes that occur during fruit development and postharvest storage (Table 4). Proteomic analyses, particularly using 2D gel electrophoresis and mass spectrometry, have identified significant alterations in the abundance of proteins related to sugar metabolism, antioxidation, allergenicity, and fruit softening [86,87,88,89,90,91,92,93]. These studies have been instrumental in characterizing the proteomic phenotypes of kiwifruit, which are crucial for understanding fruit nutritional and keeping qualities.

For example, a study conducted on the ‘Hayward’ and ‘Garmrok’ kiwifruit cultivars revealed differential expression of 90 and 106 proteins, respectively, in response to exogenous ethylene treatment [93]. This indicated significant proteome changes that potentially contribute to fruit ripening and quality traits. Another investigation into the chilling injury mechanism of hardy kiwifruit (*Actinidia arguta*) elucidated the metabolic pathways affected by low-temperature storage, highlighting the roles of proteins in mitigating stress impacts [90].

Mass spectrometry methods have been pivotal in identifying hundreds of proteins in ripe kiwifruit, shedding light on allergens and the biochemical processes involved in fruit softening. The identification of kiwellin and actinidain as allergens underscores the importance of proteomic studies in food safety and allergenicity research [93].

Moreover, the application of proteomics has not only elucidated the metabolic regulation at the protein level relevant to fruit composition and quality traits, but also provided a basis for the further exploration of gene function and improvement in kiwifruit [94,95]. Future proteomics studies are expected to advance our understanding of protein-level metabolic regulation, offering new avenues for enhancing fruit quality and postharvest shelf life through targeted breeding and biotechnological interventions.

### 3.4. Metabolomics

Metabolomic studies on kiwifruit have employed advanced techniques like gas chromatography coupled to mass spectrometry (GC-MS) and headspace solid-phase microextraction (HS-SPME) coupled to GC-MS [92,96,97,98,99], significantly deepening our understanding of the fruit’s biochemical composition and quality attributes [100,101,102]. These methods have elucidated over 500 metabolites during the development of *A. chinensis*, showcasing key alterations in sugars, organic acids, and amino acids that contribute to the fruit’s taste. The profiling extends to carotenoids, chlorophylls, vitamins, and phytohormones, painting a detailed picture of the fruit’s nutritional and biochemical landscape [103].

HS-SPME, in particular, has advanced the characterization of aroma volatile organic compounds, which are pivotal for kiwifruit flavor, identifying critical compounds like esters, alcohols, ketones, and terpenes [100,101,104,105]. This has opened new windows into understanding the appealing aroma of the fruit. Additionally, secondary metabolites such as polyphenolics, vitamin C, and carotenoids have been assessed, enriching our knowledge of kiwifruit’s health-promoting properties [106,107,108,109,110].

Integrating metabolite profiling with gene expression patterns has paved the way for a more comprehensive understanding of metabolic regulation related to fruit composition and quality traits [69,73,87,99,111]. This holistic view not only clarifies the biochemical basis of kiwifruit attributes, but also serves as a pivotal tool for breeding programs aimed at enhancing fruit quality, nutritional value, and flavor.

Key metabolites identified across these studies, such as sugar compounds (fructose, glucose), organic acids (citric acid, quinic acid), amino acids, and specific volatiles like hexanal and ethyl butanoate, offer insights into the metabolic nuances of kiwifruit quality and flavor profiles (Table 4). Furthermore, the characterization of secondary metabolites, including polyphenolics, vitamin C, and carotenoids, underscores the health-promoting attributes of kiwifruits, aligning with consumer interests in nutritious and functional foods.

By understanding the specific metabolites that contribute to kiwifruits’ desirable traits, producers and marketers can tailor their strategies to highlight these aspects, potentially leading to improved market positioning and consumer preference for kiwifruit. This approach not only aids in differentiating kiwifruits in a competitive market, but also aligns with increasing consumer interest in functional foods that offer health benefits beyond basic nutrition.

**Table 4:** Summary of recent research on kiwifruit proteomics and metabolomics.

Author et al., Year	Key Pathways Identified	Key Traits Under Study	Proteins/Metabolites Identified	Methods Used
Günther et al., 2011 [104]	Methylsulfanyl-volatiles pathways	Volatiles	Methylsulfanyl-volatiles	Headspace analysis
McGhie, 2013 [110]	Secondary metabolite components	Metabolites	Secondary metabolites	Not specified
Commisso et al., 2019 [103]	Tryptophan decarboxylase pathways	Development of kiwifruits	Tryptophan-derived metabolites	Untargeted and targeted metabolomics
Shin et al., 2020 [93]	Ethylene response pathways	Fruit ripening	Ethylene biosynthesis enzymes and cell wall-modifying proteins	Proteomic analysis
Xiong et al., 2020 [97]	Developmental stage pathways	Nutritional components	Sugars, organic acids, and amino acids	Metabolomic and transcriptomic approaches
Yu et al., 2020 [99]	Flavonoids and anthocyanin pathways	Gene analyses of kiwifruit and kiwiberry	Flavonoids and anthocyanins	Metabolomics study
Wang et al., 2021 [89]	AcMYB16 role in response to *Pseudomonas syringae* pv. *actinidiae*	Disease response	AcMYB16 and defense-related proteins	Transcriptomic and proteomic profiling
Zhang et al., 2021 [90]	Mechanisms of chilling injury	Chilling injury	Heat shock proteins and oxidative stress-related proteins	Label-free proteome techniques
Tian et al., 2021 [92]	Regulatory pathways of ripening and quality	Postharvest ripening	Ethylene-responsive proteins and ripening-associated metabolites	Proteomics and metabolomics
Rowan et al., 2021 [96]	Metabolite variability	Orchard variability of two cultivars	Primary metabolites and secondary metabolites	Metabolomics
Zhao et al., 2021 [100]	Aroma profile pathways	Aroma in three kiwifruit varieties	Volatile organic compounds (VOCs)	HS-SPME-GC-MS and GC-IMS coupled with DSA
Lan et al., 2021 [102]	Aroma chemical composition	Common kiwifruit cultivars in China	Aroma-related metabolites	Not specified
Liang et al., 2021 [107]	Physicochemical, nutritional, and bioactive properties	Pulp and peel from 15 kiwifruit cultivars	Nutritional and bioactive metabolites	-
Starowicz et al., 2022 [105]	VOCs in kiwiberries	Kiwiberries	Volatile organic compounds (VOCs)	HS-SPME/GC-MS
Choi et al., 2022 [109]	Metabolites and antioxidant activities	Green ‘Hayward’ and gold ‘Haegeum’ kiwifruits	Antioxidant metabolites	Ethylene treatment
Wang et al., 2022 [73]	Flavor formation pathways	Kiwifruit	Flavor-related metabolites	Integrative analyses of metabolome and genome-wide transcriptome
Qu et al., 2023 [86]	Potential mechanisms of SA in triggering resistance	Resistance to *Pseudomonas syringae* pv. *actinidiae*	Pathogenesis-related proteins and SA-responsive proteins	4D proteome investigation
Li et al., 2023 [91]	Amyloplast biogenesis and differentiation	Amyloplast development	Starch biosynthesis enzymes and amyloplast-specific proteins	Proteome analysis
Li et al., 2023 [98]	Auxin pathways in postharvest resistance	Postharvest resistance to Botrytis cinerea	Auxin-responsive proteins and resistance-related metabolites	Widely targeted metabolomics analysis
Wang et al., 2023 [101]	Volatile profiles of kiwifruits with soft rot	Soft rot in kiwifruits	Volatile organic compounds (VOCs)	E-nose and HS-SPME/GC–MS
Bi et al., 2023 [106]	Forchlorfenuron pathways	Kiwifruit development	Forchlorfenuron-responsive metabolites	Metabolomics
Shu et al., 2023 [69]	Major quality regulations	Red-flesh kiwifruit	Quality-related metabolites	Metabolic map
Xiong et al., 2023 [111]	Color formation pathways	Yellow-fleshed kiwifruit	Color-related metabolites	Integrative analysis of metabolome and transcriptome
Valasiadis et al., 2024 [87]	High and low dry matter pathways	Dry matter content in kiwifruit	Metabolites related to sugar and acid content	Spatiotemporal multi-omics approach
Polychroniadou et al., 2024 [88]	Calcium-related pathways	Ripening processes	Calcium-binding proteins and pectin methylesterase	Cross-omics approach

## 4. Kiwifruit Breeding

### 4.1. Conventional and Molecular Breeding

The journey of kiwifruit breeding has been marked by significant advancements, leveraging the power of multi-omics to enhance understanding and manipulation of complex traits for cultivar development. The integration of genomics, transcriptomics, proteomics, and metabolomics offers a comprehensive toolkit for dissecting the genetic and molecular bases of desirable attributes in kiwifruit, paving the way for precision breeding and the development of superior cultivars.

The breeding of kiwifruit, including species like *Actinidia arguta* (kiwiberries), has been propelled by the understanding of polyploidy and genomic selection, focusing on traits like fruit quality, yield, and stress resistance. Recent efforts have explored the effects of incorporating probabilistic versus realized relationship matrices into breeding value estimates, highlighting the complexities of chromosome inheritance and the need for models accommodating polyploidy in kiwifruits [112]. The development of new cultivars, such as ‘Hort16A’ and ‘Zesy002’, has revitalized the kiwifruit industry by introducing traits like yellow flesh and Psa tolerance, demonstrating the impact of targeted breeding programs [113].

Looking forward, the breeding of kiwifruit faces challenges and opportunities, particularly in addressing climate change, disease resistance, and the enhancement of fruit quality and nutritional value. The potential of wild *Actinidia* species, such as *A. callosa* and *A. strigosa*, for breeding cold-resistant cultivars and expanding cultivation into new regions underscores the importance of genetic diversity and exploration of underutilized genetic resources [114].

The integration of multi-omics approaches into kiwifruit breeding strategies offers unprecedented opportunities for unraveling the genetic underpinnings of complex traits. Genomic selection, facilitated by dense marker maps, enables the prediction of breeding values across different ploidy levels, enhancing selection efficiency and accelerating genetic gain [112]. Transcriptomic analyses provide insights into gene expression patterns related to fruit development, ripening, and stress responses, identifying candidate genes for targeted breeding. Proteomic and metabolomic studies further elucidate the biochemical pathways and metabolic networks that underlie fruit composition, quality, and flavor, offering markers for phenotypic selection [112].

**Figure 1:**
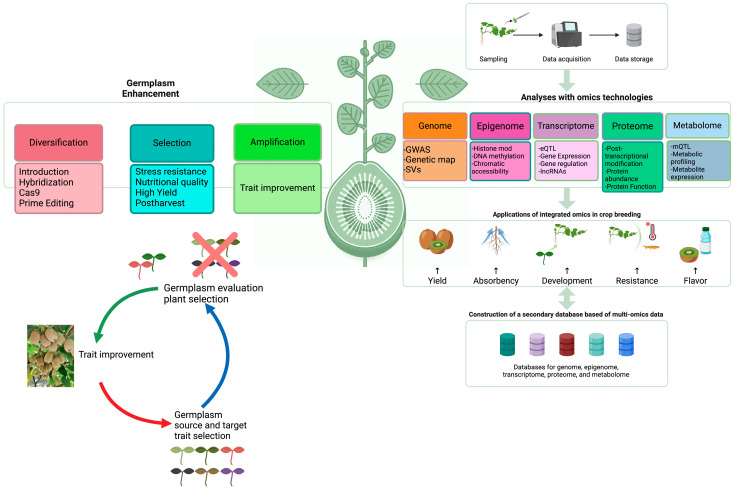
Integrative omics framework in kiwifruit breeding and trait enhancement.

The convergence of these omics technologies empowers breeders to dissect and manipulate the molecular and genetic architecture of kiwifruit with unprecedented precision. By integrating genetic, transcriptomic, proteomic, and metabolomic data, breeders can now predict the phenotypic outcomes of breeding decisions more accurately, streamline the selection process, and ultimately accelerate the development of kiwifruit cultivars with enhanced quality, resilience, and nutritional value (Figure 1).

This figure represents a comprehensive framework outlining the integration of various omics technologies in the enhancement of kiwifruit germplasm and trait improvement. The central kiwifruit illustration symbolizes the core of breeding efforts, surrounded by omics approaches: genomic, epigenomic, transcriptomic, proteomic, and metabolomic analyses. These techniques are applied to understand and manipulate complex traits, leading to improvements in yield, resistance, flavor, and other desired characteristics. On the left side, the germplasm enhancement process is depicted, highlighting the steps of diversification, selection, amplification, and evaluation for advancing kiwifruit varieties. This includes the introduction of new genetic variations through techniques such as hybridization and advanced editing tools like CRISPR-Cas9. The feedback loop emphasizes the continual cycle of trait improvement and selection. On the right, the flowchart demonstrates the process of sampling, data acquisition, and storage, culminating in the applications of integrated omics data for crop breeding. The bottom section indicates the creation of a secondary database built on multi-omics data, ensuring a holistic approach to kiwifruit breeding programs.

### 4.2. Marker-Assisted Selection

Marker-assisted selection (MAS) in kiwifruit breeding represents a pivotal advancement, leveraging the power of molecular markers to expedite the identification and incorporation of desirable traits, such as disease resistance, fruit quality, and yield into new kiwifruit cultivars. This approach has shown considerable promise, especially given the complexity of kiwifruit genetics, including high levels of heterozygosity and polyploidy across different species within the *Actinidia* genus [4]. Recent developments in SNP (single nucleotide polymorphism) genotyping arrays, such as the 135K SNP array developed for *Actinidia arguta* [52], have significantly enhanced the capabilities for genetic mapping, QTL (quantitative trait loci) analysis, and the elucidation of the genetic basis of important agronomic traits.

The integration of marker-assisted selection (MAS) into kiwifruit breeding programs signifies a pivotal advancement in the development of improved kiwifruit cultivars. MAS utilizes molecular markers closely linked to desirable traits to select plants that carry the beneficial genes, even before these traits are phenotypically observable [115]. This technique enhances the efficiency and precision of breeding programs [115,116], allowing for the rapid development of new kiwifruit varieties with enhanced qualities, such as increased disease resistance, improved fruit quality, and yield.

Recent studies have demonstrated significant progress in applying MAS for developing disease-resistant kiwifruit cultivars [47,117,118,119]. For example, SSR markers have been identified that can distinguish hybrid progeny with disease resistance [117], indicating a future where kiwifruit can be bred to withstand pathogens like Psa (*Pseudomonas syringae* pv. *actinidiae*), which has caused severe losses in the industry. By narrowing down candidate regions linked to Psa3 resistance, researchers have laid a foundation for breeding new kiwifruit cultivars that can sustainably resist this pathogen, potentially shortening the development time for disease-resistant varieties.

Moreover, the development of a high-density SNP genotyping array for kiwifruit represents a significant leap forward [52]. This array, comprising over 134,000 unique SNPs, has facilitated genetic studies and breeding applications by enabling genome-wide DNA polymorphism analysis. Such tools not only aid in the characterization of genetic diversity among kiwifruit germplasms, but also bolster the identification of quantitative trait loci (QTL) for important agronomic traits. The array’s effectiveness in distinguishing kiwifruit accessions and constructing integrated linkage maps exemplifies its utility in accelerating the breeding process through the application of genomic selection (GS) and MAS.

The future prospects for kiwifruit improvement via MAS and related genomic technologies are promising. With continued advancements in genotyping technologies and a deeper understanding of kiwifruit genetics, MAS is poised to play a crucial role in addressing challenges such as climate resilience, pest and disease resistance, and the optimization of fruit quality traits. The integration of MAS, along with other omics technologies, into breeding strategies, offers the potential to revolutionize kiwifruit breeding, making it more efficient and targeted. This will not only enhance the sustainability and productivity of kiwifruit orchards, but also meet the evolving demands of consumers for high-quality, nutritious fruit.

### 4.3. Genomic Selection

Genomic selection (GS) uses genome-wide molecular marker data to predict breeding values for quantitative traits controlled by many small-effect loci [120]. GS models are developed by associating markers dispersed across the genome with phenotype data in a training population [121]. The model is then applied to a selection population to predict genomic estimated breeding values (GEBVs) for complex polygenic traits. GS can enhance genetic gains for traits like fruit quality, yields, and quantitative disease resistance. Simulation studies determined GS in plants would provide greater genetic gains compared to MAS [122]. GS has yet to be practically implemented in kiwifruit, but would clearly benefit breeding complex fruit quality attributes, like taste, aroma, texture, and phytonutrient content.

### 4.4. Enhancing Kiwifruit Breeding through Mutation Breeding and CRISPR-Cas9 Technologies

Mutation breeding, encompassing techniques such as radiation or chemical mutagens, offers a powerful approach to generating novel trait variations by inducing DNA changes in cultivars or breeding lines. Notable examples include the irradiation of *A. deliciosa* seeds, leading to the emergence of the cultivar, which is characterized by earlier and more consistent bearing [123]. Chemically-induced mutants have displayed altered ripening behaviors and introduced novel fruit characteristics [124]. Despite the inherent risks of deleterious mutations, mutation breeding has been instrumental in creating commercially valuable new phenotypes [125]. Polyploidization, another form of mutation induction, often results in larger fruit sizes, as evidenced by Colchicine-induced tetraploids of *A. chinensis*, which exhibited fewer seeds and improved fruit quality [126]. This approach, when combined with rigorous selection, provides a valuable source of genetic diversity.

Recent studies have further advanced the application of mutation breeding in kiwifruit. For instance, the CRISPR-Cas9-mediated mutagenesis of kiwifruit BFT genes has resulted in an ever-growing but not early flowering phenotype, highlighting the potential of gene editing to introduce desirable traits without affecting the plant’s reproductive cycle [127]. Another study exploited CRISPR/Cas9 technology to induce stable hermaphroditism in a male genotype of *Actinidia chinensis* var. *chinensis*, emphasizing the use of targeted genome editing as a precise, convenient, and time-saving method compared to traditional breeding approaches [124]. These advancements demonstrate the CRISPR/Cas9 system’s utility for multiplexed gene editing, offering a robust toolkit for functional genomic research and direct applications in plant molecular breeding.

Looking ahead, mutagenesis, particularly with the integration of CRISPR/Cas9 technology, holds significant promise for future breeding programs in kiwifruit. By enabling precise modifications at the DNA level, these techniques can help overcome the limitations of traditional breeding methods, accelerating the development of kiwifruit cultivars with enhanced disease resistance, improved fruit quality, and reduced dormancy periods. As we advance, the ability to manipulate genetic material directly will undoubtedly play a critical role in shaping the next generation of kiwifruit cultivars, offering tailored solutions to the challenges posed by climate change, consumer preferences, and agricultural sustainability. This integration of mutation breeding with advanced genomic tools signifies a new era in kiwifruit breeding, where the rapid introduction of beneficial traits could significantly enhance commercial value and environmental resilience.

### 4.5. Interspecific Hybridization

Controlled hybridization between cultivated kiwifruit (*Actinidia* spp.) and wild *Actinidia* species offers a promising strategy for introgressing beneficial traits, such as disease and frost resistance, distinctive fruit-flesh coloration, improved shelf life, and enhanced flavor and sugar components. This approach has been exemplified by hybrids with *A. arguta* conferring disease and frost resistance, *A. kolomikta* derivatives introducing unique fruit-flesh coloration, *A. melanandra* hybrids improving shelf life, and *A. chrysantha* crosses increasing soluble solids [18].

A landmark in kiwifruit breeding, the cultivar “Jinyan,” resulted from crossing *A. chinensis* and *A. eriantha*, showcasing the commercial viability of interspecific hybridization [128]. This cultivar combines desirable traits, such as large fruit size, good taste, and excellent storage quality, marking a significant step forward in kiwifruit cultivar development. Furthermore, the creation of amphihaploid plants from crosses between *A. kolomikta* and other species like *A. arguta* var. *hypoleuca*, *A. polygama*, and *A. rufa* through flow cytometric analysis has provided insights into ploidy dynamics and compatibility among different *Actinidia* species [129].

Despite these successes, challenges remain in integrating desirable genes from wild species while minimizing linkage drag—whereby negative traits inadvertently accompany the desired genes, reducing overall fruit quality. Addressing these challenges requires additional backcross generations to refine and recover optimal phenotypes, a process that necessitates precise and patient breeding strategies.

The future of kiwifruit improvement lies in the wise introgression of wild *Actinidia* diversity, which can significantly enrich the gene pool. Utilizing interspecific hybridization, breeders can tap into a broader genetic base, bringing forth novel traits and cultivars that meet the growing demands for fruit quality, disease resistance, and environmental adaptability. This strategy, coupled with advanced genomic tools and a deeper understanding of *Actinidia* genetics, offers a comprehensive approach to developing superior kiwifruit varieties that are resilient, nutritious, and appealing to consumers worldwide.

### 4.6. Cisgenesis and Genome Editing

Advanced breeding techniques, like cisgenics and genome editing, accelerate precisely targeted trait modifications without foreign DNA. Cisgenesis uses genes from closely related species, thus mimicking traditional breeding. Candidate Psa resistance genes from a wild kiwifruit relative were successfully cisgenically inserted into *A. chinensis* [130]. Genome editing via CRISPR/Cas enables the precise editing of native genomic loci. CRISPR was used to mutate a citrus anthocyanin biosynthesis gene, generating a non-pigmented mutant fruit phenotype [131]. These emerging approaches enable rapid targeted trait development. However, regulatory uncertainties remain regarding commercial use, similar to transgenic GMOs. Public acceptance research is also warranted.

Kiwifruits (*Actinidia* spp.) are dioecious perennial vines with high genetic heterozygosity and long juvenile phases, posing challenges for breeding improved varieties [18,30]. However, synthetic directed evolution (SDE) offers new possibilities to enhance desired traits in kiwifruit [132,133,134]. SDE applies iterative rounds of localized sequence diversification (LSD) to target genes, coupled with selection pressure, to evolve novel genetic variants with superior phenotypes [135]. Various SDE tools can introduce LSD in plants, including CRISPR-Cas9, base editors, retrons, and EvolvR [133]. For example, CRISPR-Cas9 targeted mutagenesis of the mildew resistance locus (MLO) gene in grapes conferred resistance to powdery mildew [136]. SDE could be applied to diversify kiwifruit MLO variants and select resistant mutants on high mildew spore media. Similarly, a CRISPR base editor introduced bialaphos resistance in rice by diversifying the acetolactate synthase (ALS) gene [137]. Base editing of kiwifruit ALS could evolve herbicide resistance. A tiled CRISPR single-guide RNA library targeting the entire kiwifruit sucrose synthase gene could be delivered to induce indels via non-homologous end joining [138,139]. Regenerating shoots on high sucrose media would apply selective pressure, potentially yielding sucrose synthase variants with improved kinetic properties [140,141]. After identified through sequencing, improved alleles from SDE can be introgressed into an elite kiwifruit germplasm. Multiple SDE cycles may further optimize variants. Valuable kiwifruit traits for directed evolution include fruit quality, phytonutrient content, yield components, pest/disease resistance, and climate resilience [142]. SDE enables the rapid breeding of complex polygenic traits in diverse plant species.

## 5. Key Traits for Improvement 

Breeding kiwifruit has prioritized increasing fruit size and improving appearance to satisfy consumer preferences [40,143,144,145,146], achieving notable advancements in manipulating fruit shape, hairiness, color, and symmetry to enhance market appeal [147,148,149]. Variations in skin color, spanning green to red, and flesh color diversity, including shades of green, yellow, orange, and red, cater to diverse market needs [150,151,152]. However, challenges persist in reducing shape irregularities and deformities that affect fruit marketability, with the ideal characteristics for fruit size, shape, and color varying across different consumer demographics and applications.

In enhancing eating quality and flavor, the focus has been on achieving a harmonious balance between sugars, acids, and volatile compounds to enhance sweetness, mitigate excessive tartness, and introduce unique flavors [153,154]. This entails increasing the soluble solids content (SSC) and reducing acidity, while also developing novel volatile compounds that influence taste preferences. The pursuit of improved texture and phytonutrient levels, including vitamin C, carotenoids, and polyphenols, is informed by comprehensive evaluations involving SSC, organic acid levels, metabolites, and consumer sensory feedback, aiming to produce kiwifruit that optimally balances taste and nutritional value.

Addressing disease and pest resistance is another critical aspect, with breeding efforts focused on combating pathogens like *Pseudomonas syringae* pv. *actinidiae* (Psa), which causes canker lesions and vine dieback [51,155,156]. Advanced breeding techniques, including marker-assisted selection, cisgenics, and genome editing, are employed to incorporate resistance genes from wild kiwifruit or other species. Moreover, the breeding programs also target resistance against other pathogens, like *Botrytis cinerea* [98] and various viruses, emphasizing the selection of robust vine varieties and the adoption of suitable cultivation practices to enhance productivity and minimize losses. Root autotoxicity poses a significant challenge in kiwifruit cultivation, with Okada et al. (1000) demonstrating the inhibitory effects of kiwifruit root extracts on plant growth, contributing to replant problems [157]. Additionally, the antioxidant properties of kiwifruit have been shown to protect against oxidative stress and enhance immune responses, as reported by Hunter et al. [158]. Moreover, modern biotechnology has been applied to enhance kiwifruit’s resistance to various biotic and abiotic stresses by manipulating stress tolerance genes, as noted by Xu et al. [159].

Managing abiotic factors influencing yield and quality are major objectives in breeding programs. Kiwifruit faces various abiotic threats that impact its growth, development, and postharvest quality. Yin et al. (2012) examined the differential expression of *AdERF* genes in kiwifruit in response to postharvest abiotic stress, highlighting the complex regulatory mechanisms involved in stress responses [160]. Salt stress, a significant abiotic threat, was studied by Yuan et al. [161] who found that ‘Watt’ kiwifruit seedlings exhibited stronger tolerance to salt stress compared to ‘Hayward’, with *WRKY* genes playing a crucial role in this response. Jing et al. [162] conducted a genome-wide identification of *WRKY* transcription factors in kiwifruits, analyzing their expression in response to both biotic and abiotic stresses, thus providing valuable insights into the plant’s defense mechanisms. Drought stress, another critical abiotic threat, was mitigated by exogenous melatonin through the activation of the ascorbic acid–glutathione (*AsA-GSH*) cycle, carotenoid biosynthesis, and protective enzyme systems, as shown by Zhao et al. [163].

Yield improvement is a complex endeavor, entailing the analysis of factors such as the number of fruits per vine, fruit size distribution, and vine growth traits, including budbreak timing, leaf area, and flowering duration. Research into genotype–phenotype relationships for these yield components is key to enabling genomic selection for better productivity [164]. The development of optimal vine architectures that are tailored to specific growing environments and compatible with mechanical harvesting systems is also vital for ensuring consistent and high yields in the face of biotic and abiotic stresses.

The shelf life and storage ability of kiwifruit are crucial for extending market reach [165], with breeding targets focusing on enhancing fruit firmness, reducing ethylene production to slow down ripening, and improving resistance to physical damage, water loss, chilling injury, or pathogens. Significant research shows that kiwifruit genotypes with thicker, waxier peels tend to maintain quality for longer durations in storage [166]. Peel morphological traits, including thicker cuticle [167], reduced lenticel density [168], and higher natural epicuticular wax [169], are associated with lower transpiration, respiration, and ethylene production. This in turn slows metabolic processes tied to ripening and senescence. The genetic and biochemical factors contributing to enhanced peel properties remain poorly understood. Omics profiling during storage phases helps uncover the molecular factors that control postharvest qualities, guiding the breeding of vines that produce fruits with durable skins, slow ripening characteristics, and enhanced disease tolerance.

Lastly, the adaptability of kiwifruit to varied environmental conditions is paramount, especially in the context of climate change. Breeding programs increasingly rely on wild germplasm to introduce traits conducive to environmental resilience, such as late budbreak and early growth cessation. The development of varieties that can withstand wind, hail, and flooding is critical, necessitating a deep understanding of genotype–environment interactions through multi-location field trials to direct breeding efforts for regional or global production adaptability.

## 6. Conclusions and Future Directions 

In the pursuit of enhancing kiwifruit breeding, future efforts should emphasize the expanded collection and evaluation of *Actinidia* germplasm, encompassing both wild species and progenitors of current cultivars. This endeavor is vital for enriching the genetic diversity accessible for breeding, aiming to systematically characterize phenotypic and genotypic attributes across varied environments. Such comprehensive analyses will facilitate the identification of valuable traits within the gene pool.

The integration of omics-based strategies in breeding programs is anticipated to revolutionize the selection process. By harnessing multi-omics data through advanced bioinformatics and systems biology, breeders can improve predictions of genomic estimated breeding values, thus refining the selection of superior parental lines for complex fruit quality attributes.

Speed breeding methodologies are gaining traction as a means to expedite breeding cycles, employing techniques like rapid generation turnover in controlled environments, LED lighting manipulation, and tissue culture. These approaches promise to increase the frequency of breeding cycles annually, accelerating the pace of genetic improvement.

Genome editing, particularly through CRISPR and other targeted modification techniques, holds immense promise for swiftly incorporating desirable traits or achieving precise genetic enhancements without the drawbacks of linkage drag or random mutations. However, the potential off-target effects and regulatory considerations present ongoing challenges.

The quest for fruit quality improvements remains a central focus, with ongoing breeding programs striving to enhance flavor, texture, appearance, phytonutrient content, and storage longevity. The development of novel fruit varieties showcasing unique colors, shapes, sizes, and flavor profiles is also anticipated.

Enhancing disease resistance is a critical objective, aiming to integrate resistance genes from wild germplasm to develop cultivars with robust defenses against Psa, fungal infections, viruses, and other pathogens, thereby supporting sustainable production practices.

Moreover, breeding efforts are directed toward increasing the yield by developing vines with optimal architectural, flowering, and growth traits, alongside improved yield components, like fruit number and size distribution, to boost overall productivity.

In conclusion, the kiwifruit industry stands to benefit significantly from ongoing and future breeding initiatives. The rich diversity of *Actinidia* germplasm, coupled with expanding omics resources, is paving the way for the development of superior cultivars. The integration of traditional and modern breeding techniques continues to drive improvements in fruit quality, yield, and disease resistance, underscoring the kiwifruit’s status as a nutritionally and economically important crop. The continued exploration and characterization of genetic resources, alongside the adoption of high-throughput phenotyping, advanced genomic tools, and innovative breeding techniques, are expected to sustain and enhance the kiwifruit’s global significance.

## Figures and Tables

**Table 3:** Detailed overview of kiwifruit transcriptomic research: pathways, traits, genes, and methodologies.

Author et al., Year	Key Pathways Identified	Key Traits Under Study	Genes/Markers Identified	Methods Used
Li et al., 2015 [78]	Phytohormones, sugars, starch, and L-ascorbic acid metabolism	Fruit quality and anthocyanin accumulation	-	Transcriptome sequencing and gene expression profiling
Kamboj et al., 2020 [79]	Molecular polymorphism	Diversity analysis and genotype identification	RAPD markers	Diversity analysis and genotype identification
Zhang et al., 2020 [80]	Ripening-related ester biosynthesis	Fruit ripening and ester content	*AdFAD1*, *AdALDH2*, *AdAT17*, *AdNAC5*, *AdDof4*	Co-expression network analysis
Wu et al., 2020 [63]	Phytohormone pathways	Fruit development	*CPPU*, *IAA*	Transcriptome sequencing and phytohormone analysis
Zambounis et al., 2020 [64]	Pathogen response pathways	Disease resistance	*PR*, *CHI*	RNA sequencing-based transcriptome analysis
Qiu et al., 2020 [76]	Coloration and quality pathways	Coloration and fruit quality	*MYB*, *DFR*	Transcriptome and metabolome analyses
Lin et al., 2020 [66]	Hydrogen sulfide signaling pathways	Ripening delay	*PG*, *EXP*	Transcriptome analysis
Liang et al., 2020 [70]	Phenolic synthesis pathways	Fruit development and ripening	*CHS*, *F3H*	Gene expression profiling
Salazar et al., 2021 [65]	Ethylene signaling pathways	Fruit ripening	*ETR*, *EIN*	Transcriptome analysis
Brian et al., 2021 [81]	Floral bud and flower development, fruit development and maturation, and ethylene-induced fruit ripening	Transcriptional control of floral bud, flower, and fruit development, and ethylene response	*AP2/ERF*, *bHLH*, *MYB*	Network analysis and transcriptome profiling
Yang et al., 2021 [82]	Nitric oxide regulation during fruit softening	Fruit ripening inhibition	*Genes related to nitric oxide regulation*	Transcriptome profiling
Sun et al., 2021 [83]	Cellulose degradation, trehalose synthesis, starch degradation, and cold response	Freezing tolerance and low-temperature response	*beta-GC*, *TPS5*, *BAM3.1*, *CBF3*, *MYC2*, *MYB44*	Transcriptome profiling and WGCNA
Tu et al., 2021 [72]	Chlorophyll degradation pathways	Chlorophyll degradation	*CAB*, *SGR*	Transcriptome analysis
Burdon et al., 2021 [68]	Maturation pathways	Fruit maturation	*MYB*, *MADS-box*	Transcriptomic analysis
Salazar et al., 2021 [77]	Transcriptomic pathways	Tissue-specific transcriptomics	*AP2/ERF*, *bHLH*	De novo transcriptome sequencing
Huan et al., 2021 [74]	Starch degradation and fermentation pathways	Alcoholic off-flavor development	*AMY*, *PDC*	Transcriptome analysis
Wang et al., 2022 [73]	Flavor formation pathways	Flavor formation	*LOX*, *ADH*	Metabolome and genome-wide transcriptome analyses
Tao et al., 2022 [84]	Stress responses, phytohormone signal transduction, and plant growth and development	TIFY gene family functions	*TIFY gene family (JAZ*, *ZML*, *TIFY*, *PPD)*	Genome-wide identification and characterization
Xiong et al., 2022	mRNA editing post-pathogen infection	Pathogen stress response	*MORF* genes	Genome-wide analysis
Jia et al., 2022 [85]	Flavonoid biosynthesis and chalcone synthase gene family	Parthenocarpy in seedless kiwifruit	Chalcone synthase (CHS) gene family	Full-length transcriptome sequencing
Shu et al., 2023 [69]	Metabolic regulatory networks during development and ripening	Kiwifruit quality improvement	Various genes related to metabolic regulation	Metabolomic and transcriptomic analyses
Niu et al., 2023 [67]	Phenolic synthesis and phytohormone pathways	Chilling injury mitigation	*PAL*, *PR1*	Transcriptome analysis
Shu et al., 2023 [69]	Metabolic pathways	Fruit quality	*ANS*, *DFR*	Metabolic mapping
Guo et al., 2024 [71]	Comparative gene expression pathways	Gene expression differences	*WRKY*, *NAC*	Comparative transcriptome analysis
Wang, Y.; et al. 2024 [75]	Cell wall metabolism pathways	Postharvest softening	*XTH*, *PME*	Transcriptomic analysis
Yang et al., 2024 [62]	Metabolomic and transcriptomic pathways	Postharvest ripening	*ACS*, *ACO*	Integrated metabolomic and transcriptomic analyses

## Data Availability

No new data were created or analyzed in this study. Data sharing does not apply to this article.

## References

[1] Hazarika B., Angami T., Parthasarathy V. (2022). Fruits: Tropical and Subtropical, Kiwifruit.

[2] Huang H. (2016). Kiwifruit: The Genus Actinidia.

[3] Datson P., Ferguson A. (2011). Actinidia. Wild Crop Relatives: Genomic and Breeding Resources: Tropical and Subtropical Fruits.

[4] Qi B., Wang F., Ye K., Mo Q., Gong H., Liu P., Jiang Q., Li J. (2023). Genetic Diversity of 52 Species of Kiwifruit (*Actinidia chinensis* Planch.). Horticulturae.

[5] Hanley Z. (2018). Kiwifruit (*Actinidia* spp.) breeding. Advances in Plant Breeding Strategies: Fruits.

[6] Ferguson A.R. (2011). Kiwifruit: A botanical review. Horticultural Reviews.

[7] Huang H., Ferguson A.R. (2001). Kiwifruit in China. N. Z. J. Crop Hortic. Sci..

[8] Huang H.-W., Ferguson A. *Actinidia* in China: Natural diversity, phylogeographical evolution, interspecific gene flow and kiwifruit cultivar improvement. Proceedings of the VI International Symposium on Kiwifruit.

[9] Ferguson A.R., Huang H., Costa G. (2023). History of Kiwifruit: Evolution of a Global Crop. Kiwifruit: Botany, Production and Uses.

[10] Testolin R. Kiwifruit (*Actinidia* spp.) in Italy: The history of the industry, international scientific cooperation and recent advances in genetics and breeding. Proceedings of the VIII International Symposium on Kiwifruit.

[11] Tait P.R., Rutherford P., Driver T., Li X., Saunders C.M., Dalziel P.C., Guenther M. (2018). Consumer Insights and Willingness to Pay for Attributes: Kiwifruit in Shanghai, China.

[12] Henare S.J. (2016). The nutritional composition of kiwifruit (*Actinidia* spp.). Nutritional Composition of Fruit Cultivars.

[13] Satpal D., Kaur J., Bhadariya V., Sharma K. (2021). *Actinidia deliciosa* (Kiwi fruit): A comprehensive review on the nutritional composition, health benefits, traditional utilization, and commercialization. J. Food Process. Preserv..

[14] Richardson D.P., Ansell J., Drummond L.N. (2018). The nutritional and health attributes of kiwifruit: A review. Eur. J. Nutr..

[15] Drummond L. (2013). The composition and nutritional value of kiwifruit. Adv. Food Nutr. Res..

[16] Wang R., Li X., Sun M., Xue C., Korban S.S., Wu J. (2023). Genomic insights into domestication and genetic improvement of fruit crops. Plant Physiol..

[17] Testolin R., McNeilage M.A. (2023). Genetic Improvement, Kiwifruit Genome and Dioecy. Kiwifruit: Botany, Production and Uses.

[18] Huang H., Liu Y. (2014). Natural hybridization, introgression breeding, and cultivar improvement in the genus *Actinidia*. Tree Genet. Genomes.

[19] Pathak R., Pant V., Negi V.S., Bhatt I.D., Belwal T. (2023). Introduction to Himalayan region and wild edible diversity. Himalayan Fruits and Berries.

[20] Cuong N.M., Soejarto D.D., Li J. (2007). A taxonomic revision of Actinidiaceae of Vietnam. Blumea-Biodivers. Evol. Biogeogr. Plants.

[21] Xie Q., Zhang H., Yan F., Yan C., Wei S., Lai J., Wang Y., Zhang B. (2019). Morphology and molecular identification of twelve commercial varieties of kiwifruit. Molecules.

[22] Testolin R. Kiwifruit breeding: From the phenotypic analysis of parents to the genomic estimation of their breeding value (GEBV). Proceedings of the VII International Symposium on Kiwifruit.

[23] Li D., Liu Y., Zhong C., Huang H. (2010). Morphological and cytotype variation of wild kiwifruit (*Actinidia chinensis* complex) along an altitudinal and longitudinal gradient in central-west China. Bot. J. Linn. Soc..

[24] Wang Y.-C., Zhang L., Man Y.-P., Li Z.-Z., Qin R. (2012). Phenotypic characterization and simple sequence repeat identification of red-fleshed kiwifruit germplasm accessions. HortScience.

[25] Leontowicz H., Leontowicz M., Latocha P., Jesion I., Park Y.-S., Katrich E., Barasch D., Nemirovski A., Gorinstein S. (2016). Bioactivity and nutritional properties of hardy kiwi fruit *Actinidia arguta* in comparison with *Actinidia deliciosa* ‘Hayward’ and *Actinidia eriantha* ‘Bidan’. Food Chem..

[26] Ferguson A. Kiwifruit cultivars: Breeding and selection. Proceedings of the IV International Symposium on Kiwifruit.

[27] Pinto T., Vilela A. (2018). Kiwifruit, a botany, chemical and sensory approach a review. Adv. Plants Agric. Res..

[28] Hale I., Melo A., Gustafson H. (2018). Sex-linked molecular markers for two cold-hardy kiwifruit species, *Actinidia arguta* and *A. kolomikta*. Eur. J. Hortic. Sci..

[29] Wang S., Jiang Z., Zhang Z., Gong J., Huang H. Exploration of *Actinidia* genetic resources and development of kiwifruit industry in China. Proceedings of the V International Symposium on Kiwifruit.

[30] Ferguson A.R., Huang H. (2007). Genetic resources of kiwifruit: Domestication and breeding. Horticultural Reviews.

[31] Huang H. (2011). Plant diversity and conservation in China: Planning a strategic bioresource for a sustainable future. Bot. J. Linn. Soc..

[32] Huang H. (2010). Ex situ plant conservation: A key role of Chinese botanic gardens in implementing China’s strategy for plant Conservation. BGjournal.

[33] Wang Y.-X., Zhou W.-Y., Zhang W.-H., Wu W.-W., Zhang X.-J., Yu Y.-H. (2021). Genetic structure analysis of 85 kiwifruit varieties (lines) and wild relatives by SCoT molecular markers. J. Fruit Sci..

[34] Jamali Anjelani S., Ghasemnezhad M., Samizadeh H., Hamidoghli Y. (2020). Validation of Some Molecular Markers in Sex Determination in Different Kiwifruit Genotypes from Open Pollination. J. Plant Prod. Res..

[35] Hu G., Jiang Q., Wang Z., Li Z., Liao W., Shen D., Zhong C. (2022). Genetic Diversity Analysis and Core Collection Construction of the *Actinidia chinensis* Complex (Kiwifruit) Based on SSR Markers. Agronomy.

[36] Liao G., Xu X., Huang C., Qu X., Jia D. (2023). A novel early maturing kiwifruit (*Actinidia eriantha*) cultivar. N. Z. J. Crop Hortic. Sci..

[37] Chłosta I., Kwolek D., Sliwinska E., Góralski G., Popielarska-Konieczna M. (2021). Sex-linked molecular markers identify female lines in endosperm-derived kiwifruit callus and in regenerants. Plants.

[38] Cheng J., Guo W., Du R., Zhou Y. (2022). Optical properties of different kiwifruit cultivars (*Actinidia deliciosa* and *Actinidia chinensis*) and their correlation with internal quality. Infrared Phys. Technol..

[39] Tilahun S., Choi H.R., Park D.S., Lee Y.M., Choi J.H., Baek M.W., Hyok K., Park S.M., Jeong C.S. (2020). Ripening quality of kiwifruit cultivars is affected by harvest time. Sci. Hortic..

[40] Figiel-Kroczyńska M., Ochmian I., Lachowicz S., Krupa-Małkiewicz M., Wróbel J., Gamrat R. (2021). *Actinidia* (mini kiwi) fruit quality in relation to summer cutting. Agronomy.

[41] Nadarajan J., Esfandiari A., Mathew L., Divinagracia J., Wiedow C., Morgan E. (2023). Development, Management and Utilization of a Kiwifruit (*Actinidia* spp.) In Vitro Collection: A New Zealand Perspective. Plants.

[42] Debenham M., Pathirana R. (2021). Establishment and management of an in vitro repository of kiwifruit (*Actinidia* spp.) germplasm. Meta-Topolin: A Growth Regulator for Plant Biotechnology and Agriculture.

[43] Huang H., Wang Y., Zhang Z., Jiang Z., Wang S. (2004). *Actinidia* germplasm resources and kiwifruit industry in China. HortScience.

[44] Wang F.M., Mo Q.H., Ye K.Y., Gong H.J., Qi B.B., Liu P.P., Jiang Q.S., Li J.W. (2020). Evaluation of the wild *Actinidia* germplasm for resistance to *Pseudomonas syringae* pv. actinidiae. Plant Pathol..

[45] Crowhurst R.N., Gleave A.P., MacRae E.A., Ampomah-Dwamena C., Atkinson R.G., Beuning L.L., Bulley S.M., Chagne D., Marsh K.B., Matich A.J. (2008). Analysis of expressed sequence tags from *Actinidia*: Applications of a cross species EST database for gene discovery in the areas of flavor, health, color and ripening. BMC Genom..

[46] Lu X.-M., Man Y.-P., Lei R., Liu Y., Wu J.-H., Wang Y.-C. (2022). Structural analysis of *Actinidia arguta* natural populations and preliminary application in association mapping of fruit traits. Sci. Hortic..

[47] Guido C. (2024). Molecular Markers and Allele Mining in Kiwifruit Breeding. Allele Mining for Genomic Designing of Fruit Crops.

[48] Popowski E., Thomson S.J., Knäbel M., Tahir J., Crowhurst R.N., Davy M., Foster T.M., Schaffer R.J., Tustin D.S., Allan A.C. (2021). Construction of a high-density genetic map for hexaploid kiwifruit (*Actinidia chinensis* var. deliciosa) using genotyping by sequencing. G3.

[49] Zhang Q., Liu C., Liu Y., VanBuren R., Yao X., Zhong C., Huang H. (2015). High-density interspecific genetic maps of kiwifruit and the identification of sex-specific markers. DNA Res..

[50] Li S., Wang R., Lin M., Gu H., Li Y., Zhang M., Feng X., Qi X. (2023). Construction of a High-Density Genetic Map and QTL Mapping of Growth Traits in Kiwifruit. https://www.researchsquare.com/article/rs-2983542/v1.

[51] Tahir J., Brendolise C., Hoyte S., Lucas M., Thomson S., Hoeata K., McKenzie C., Wotton A., Funnell K., Morgan E. (2020). QTL mapping for resistance to cankers induced by *Pseudomonas syringae* pv. Actinidiae (psa) in a tetraploid *Actinidia chinensis* kiwifruit population. Pathogens.

[52] Wang R., Xing S., Bourke P.M., Qi X., Lin M., Esselink D., Arens P., Voorrips R.E., Visser R.G., Sun L. (2023). Development of a 135K SNP genotyping array for *Actinidia arguta* and its applications for genetic mapping and QTL analysis in kiwifruit. Plant Biotechnol. J..

[53] Li S.-K., Wang R., Qi X.-J. (2022). Recent advances in research on the molecular markers, genetic map and QTL mapping in kiwifruit. J. Fruit Sci..

[54] Yue J., Liu J., Tang W., Wu Y.Q., Tang X., Li W., Yang Y., Wang L., Huang S., Fang C. (2020). Kiwifruit Genome Database (KGD): A comprehensive resource for kiwifruit genomics. Hortic. Res..

[55] Yue J., Chen Q., Zhang S., Lin Y., Ren W., Li B., Wu Y., Wang Y., Zhou Y., Liu Y. (2024). Origin and evolution of the kiwifruit Y chromosome. Plant Biotechnol. J..

[56] Akagi T., Varkonyi-Gasic E., Shirasawa K., Catanach A., Henry I.M., Mertten D., Datson P., Masuda K., Fujita N., Kuwada E. (2023). Recurrent neo-sex chromosome evolution in kiwifruit. Nat. Plants.

[57] Pilkington S.M., Crowhurst R., Hilario E., Nardozza S., Fraser L., Peng Y., Gunaseelan K., Simpson R., Tahir J., Deroles S.C. (2018). A manually annotated *Actinidia chinensis* var. chinensis (kiwifruit) genome highlights the challenges associated with draft genomes and gene prediction in plants. BMC Genom..

[58] Xia H., Deng H., Li M., Xie Y., Lin L., Zhang H., Luo X., Lv X., Wang J., Liang D. (2023). Chromosome-scale genome assembly of a natural diploid kiwifruit (*Actinidia chinensis* var. deliciosa). Sci. Data.

[59] Huang S., Ding J., Deng D., Tang W., Sun H., Liu D., Zhang L., Niu X., Zhang X., Meng M. (2013). Draft genome of the kiwifruit *Actinidia chinensis*. Nat. Commun..

[60] Wu H., Ma T., Kang M., Ai F., Zhang J., Dong G., Liu J. (2019). A high-quality *Actinidia chinensis* (kiwifruit) genome. Hortic. Res..

[61] Yao X., Wang S., Wang Z., Li D., Jiang Q., Zhang Q., Gao L., Zhong C., Huang H., Liu Y. (2022). The genome sequencing and comparative analysis of a wild kiwifruit *Actinidia eriantha*. Mol. Hortic..

[62] Yang H., Zhang X., Wu R., Tang X., Yang Y., Fan X., Gong H., Grierson D., Yin X., Li J. (2024). Integrated metabolomic and transcriptomic analyses provide comprehensive new insights into the mechanism of chitosan delay of kiwifruit postharvest ripening. Postharvest Biol. Technol..

[63] Wu L., Lan J., Xiang X., Xiang H., Jin Z., Khan S., Liu Y. (2020). Transcriptome sequencing and endogenous phytohormone analysis reveal new insights in CPPU controlling fruit development in kiwifruit (*Actinidia chinensis*). PLoS ONE.

[64] Zambounis A., Ganopoulos I., Valasiadis D., Karapetsi L., Madesis P. (2020). RNA sequencing-based transcriptome analysis of kiwifruit infected by *Botrytis cinerea*. Physiol. Mol. Plant Pathol..

[65] Salazar J., Zapata P., Silva C., González M., Pacheco I., Bastías M., Meneses C., Jorquera C., Moreno I., Shinya P. (2021). Transcriptome analysis and postharvest behavior of the kiwifruit ‘*Actinidia deliciosa*’ reveal the role of ethylene-related phytohormones during fruit ripening. Tree Genet. Genomes.

[66] Lin X., Yang R., Dou Y., Zhang W., Du H., Zhu L., Chen J. (2020). Transcriptome analysis reveals delaying of the ripening and cell-wall degradation of kiwifruit by hydrogen sulfide. J. Sci. Food Agric..

[67] Niu Y., Ye L., Wang Y., Shi Y., Liu Y., Luo A. (2023). Transcriptome analysis reveals salicylic acid treatment mitigates chilling injury in kiwifruit by enhancing phenolic synthesis and regulating phytohormone signaling pathways. Postharvest Biol. Technol..

[68] Burdon J., Martin P., Ireland H., Schaffer R., McAtee P., Boldingh H., Nardozza S. (2021). Transcriptomic analysis reveals differences in fruit maturation between two kiwifruit cultivars. Sci. Hortic..

[69] Shu P., Zhang Z., Wu Y., Chen Y., Li K., Deng H., Zhang J., Zhang X., Wang J., Liu Z. (2023). A comprehensive metabolic map reveals major quality regulations in red-flesh kiwifruit (*Actinidia chinensis*). New Phytol..

[70] Liang D., Deng H., Deng Q., Lin L., Lv X., Wang J., Wang Z., Xiong B., Zhao X., Xia H. (2020). Dynamic changes of phenolic compounds and their associated gene expression profiles occurring during fruit development and ripening of the Donghong kiwifruit. J. Agric. Food Chem..

[71] Guo L., Yan K., Li D., Li W. (2024). Comparative transcriptome analysis revealed gene expression differences in fruits between two *Actinidia chinensis* cultivars. All Life.

[72] Tu M.-Y., Wu Y.-Y., Li J., Chen D., Jiang G.-L., Song H.-Y., Yin X.-R., Liu X.-F., Li M.-Z., Sun S.-X. (2021). Transcriptome analysis reveals the roles of chlorophyll a/b-binding proteins (CABs) and stay-green (SGR) in chlorophyll degradation during fruit development in kiwifruit. N. Z. J. Crop Hortic. Sci..

[73] Wang R., Shu P., Zhang C., Zhang J., Chen Y., Zhang Y., Du K., Xie Y., Li M., Ma T. (2022). Integrative analyses of metabolome and genome-wide transcriptome reveal the regulatory network governing flavor formation in kiwifruit (*Actinidia chinensis*). New Phytol..

[74] Huan C., Du X., Wang L., Kebbeh M., Li H., Yang X., Shen S., Zheng X. (2021). Transcriptome analysis reveals the metabolisms of starch degradation and ethanol fermentation involved in alcoholic off-flavour development in kiwifruit during ambient storage. Postharvest Biol. Technol..

[75] Wang Y., Niu Y., Ye L., Shi Y., Luo A. (2024). Transcriptomic analysis reveals ozone treatment delays kiwifruit postharvest softening by modulating cell wall metabolism. J. Food Sci..

[76] Qiu W., Su W., Cai Z., Dong L., Li C., Xin M., Fang W., Liu Y., Wang X., Huang Z. (2020). Combined analysis of transcriptome and metabolome reveals the potential mechanism of coloration and fruit quality in yellow and purple *Passiflora edulis* Sims. J. Agric. Food Chem..

[77] Salazar J.A., Vergara-Pulgar C., Jorquera C., Zapata P., Ruiz D., Martínez-Gómez P., Infante R., Meneses C. (2021). De novo transcriptome sequencing in kiwifruit (*Actinidia chinensis* var. deliciosa (A Chev) Liang et Ferguson) and development of tissue-specific transcriptomic resources. Agronomy.

[78] Li W., Liu Y., Zeng S., Xiao G., Wang G., Wang Y., Peng M., Huang H. (2015). Gene expression profiling of development and anthocyanin accumulation in kiwifruit (*Actinidia chinensis*) based on transcriptome sequencing. PLoS ONE.

[79] Kamboj A., Kharb P., Jhilta A., Singh R. (2020). Genotype identification and diversity analysis in Kiwifruit (*Actinidia* spp.) using RAPD markers. bioRxiv.

[80] Zhang A., Zhang Q., Li J., Gong H., Fan X., Yang Y., Liu X., Yin X. (2020). Transcriptome co-expression network analysis identifies key genes and regulators of ripening kiwifruit ester biosynthesis. BMC Plant Biol..

[81] Brian L., Warren B., McAtee P., Rodrigues J., Nieuwenhuizen N., Pasha A., David K.M., Richardson A., Provart N.J., Allan A.C. (2021). A gene expression atlas for kiwifruit (*Actinidia chinensis*) and network analysis of transcription factors. BMC Plant Biol..

[82] Yang R., Lin X., Dou Y., Zhang W., Du H., Wan C., Chen J., Zhang L., Zhu L. (2021). Transcriptome profiling of postharvest kiwifruit in response to exogenous nitric oxide. Sci. Hortic..

[83] Sun S., Lin M., Qi X., Chen J., Gu H., Zhong Y., Sun L., Muhammad A., Bai D., Hu C. (2021). Full-length transcriptome profiling reveals insight into the cold response of two kiwifruit genotypes (*A. arguta*) with contrasting freezing tolerances. BMC Plant Biol..

[84] Tao J., Jia H., Wu M., Zhong W., Jia D., Wang Z., Huang C. (2022). Genome-wide identification and characterization of the TIFY gene family in kiwifruit. BMC Genom..

[85] Jia Y., Wu Y.-P., Wang F.-W., Zhang L., Yu G., Wang Y.-L., Zhang Y. (2022). Full-length transcriptome sequencing analysis and characterization of gene isoforms involved in flavonoid biosynthesis in the seedless kiwifruit cultivar ‘Chengxiang’ (*Actinidia arguta*). Diversity.

[86] Qu D., Yan F., Zhang Y., Huang L. (2023). A 4D Proteome Investigation of the Potential Mechanisms of SA in Triggering Resistance in Kiwifruit to *Pseudomonas syringae* pv. actinidiae. Int. J. Mol. Sci..

[87] Valasiadis D., Kollaros M.G., Michailidis M., Polychroniadou C., Tanou G., Bazakos C., Molassiotis A. (2024). Wide-characterization of high and low dry matter kiwifruit through spatiotemporal multi-omic approach. Postharvest Biol. Technol..

[88] Polychroniadou C., Michailidis M., Samiotaki M., Adamakis I.-D.S., Giannoutsou E., Skodra C., Karagiannis E., Bazakos C., Molassiotis A., Tanou G. (2024). Understanding the effect of calcium in kiwifruit ripening and establishment of early and late response mechanisms through a cross-omics approach. Postharvest Biol. Technol..

[89] Wang X., Li Y., Liu Y., Zhang D., Ni M., Jia B., Heng W., Fang Z., Zhu L.-w., Liu P. (2021). Transcriptomic and Proteomic Profiling Reveal the Key Role of AcMYB16 in the Response of *Pseudomonas syringae* pv. actinidiae in Kiwifruit. Front. Plant Sci..

[90] Zhang L., Wu C.L., Yang P., Wang Y.C., Zhang L.L., Yang X.Y. (2021). Chilling injury mechanism of hardy kiwifruit (*Actinidia arguta*) was revealed by proteome of label-free techniques. J. Food Biochem..

[91] Li A., Lin J., Zeng Z., Deng Z., Tan J., Chen X., Ding G., Zhu M., Xu B., Atkinson R.G. (2023). The kiwifruit amyloplast proteome (kfALP): A resource to better understand the mechanisms underlying amyloplast biogenesis and differentiation. Plant J..

[92] Tian X., Zhu L., Yang N., Song J., Zhao H., Zhang J., Ma F., Li M. (2021). Proteomics and metabolomics reveal the regulatory pathways of ripening and quality in post-harvest kiwifruits. J. Agric. Food Chem..

[93] Shin M.H., Muneer S., Kim Y.-H., Lee J.J., Bae D.W., Kwack Y.-B., Kumarihami H.P.C., Kim J.G. (2020). Proteomic analysis reveals dynamic regulation of fruit ripening in response to exogenous ethylene in kiwifruit cultivars. Hortic. Environ. Biotechnol..

[94] Lalrinmawii, Mir H., Perveen N. (2023). Recent Advances in the Use of Molecular Markers for Fruit Crop Improvement. Molecular Marker Techniques: A Potential Approach of Crop Improvement.

[95] Yang F., Zhao R., Suo J., Ding Y., Tan J., Zhu Q., Ma Y. (2024). Understanding quality differences between kiwifruit varieties during softening. Food Chem..

[96] Rowan D., Boldingh H., Cordiner S., Cooney J., Hedderley D., Hewitt K., Jensen D., Pereira T., Trower T., McGhie T. (2021). Kiwifruit Metabolomics—An Investigation of within Orchard Metabolite Variability of Two Cultivars of *Actinidia chinensis*. Metabolites.

[97] Xiong Y., Yan P., Du K., Li M., Xie Y., Gao P. (2020). Nutritional component analyses of kiwifruit in different development stages by metabolomic and transcriptomic approaches. J. Sci. Food Agric..

[98] Li Z.-X., Yang S., Wang X., Liao Q.-H., Zhang W.-L., Liu J., Liu G.-H., Tang J.-M. (2023). Widely targeted metabolomics analysis reveals the effect of exogenous auxin on postharvest resistance to Botrytis cinerea in kiwifruit (*Actinidia chinensis* L.). Postharvest Biol. Technol..

[99] Yu M., Man Y., Lei R., Lu X., Wang Y. (2020). Metabolomics study of flavonoids and anthocyanin-related gene analysis in kiwifruit (*Actinidia chinensis*) and kiwiberry (*Actinidia arguta*). Plant Mol. Biol. Report..

[100] Zhao Y., Zhan P., Tian H.-L., Wang P., Lu C., Tian P., Zhang Y.-Y. (2021). Insights into the aroma profile in three kiwifruit varieties by HS-SPME-GC-MS and GC-IMS coupled with DSA. Food Anal. Methods.

[101] Wang Y., Wang D., Lv Z., Zeng Q., Fu X., Chen Q., Luo Z., Luo C., Wang D., Zhang W. (2023). Analysis of the volatile profiles of kiwifruits experiencing soft rot using E-nose and HS-SPME/GC–MS. LWT.

[102] Lan T., Gao C., Yuan Q., Wang J., Zhang H., Sun X., Lei Y., Ma T. (2021). Analysis of the aroma chemical composition of commonly planted kiwifruit cultivars in China. Foods.

[103] Commisso M., Negri S., Bianconi M., Gambini S., Avesani S., Ceoldo S., Avesani L., Guzzo F. (2019). Untargeted and targeted metabolomics and tryptophan decarboxylase in vivo characterization provide novel insight on the development of kiwifruits (*Actinidia deliciosa*). Int. J. Mol. Sci..

[104] Günther C.S., Matich A.J., Marsh K.B., Nicolau L. (2011). Development of a quantitative method for headspace analysis of methylsulfanyl-volatiles from kiwifruit tissue. Food Res. Int..

[105] Starowicz M., Błaszczak W., Ciska E., Latocha P. (2022). Characterization of volatile organic compounds in kiwiberries (*Actinidia arguta*) exposed to high hydrostatic pressure processing by HS-SPME/GC-MS. Molecules.

[106] Bi Y., Qiao C., Han L., Xie H., Xu Y., Wu D., Zhuang M., Lv X., Cao M. (2023). Key metabolites and mechanistic insights in forchlorfenuron controlling kiwifruit development. Food Res. Int..

[107] Liang J., Ren Y., Wang Y., Han M., Yue T., Wang Z., Gao Z. (2021). Physicochemical, nutritional, and bioactive properties of pulp and peel from 15 kiwifruit cultivars. Food Biosci..

[108] Sanz V., López-Hortas L., Torres M., Domínguez H. (2021). Trends in kiwifruit and byproducts valorization. Trends Food Sci. Technol..

[109] Choi H.R., Baek M.W., Cheol L.H., Jeong C.S., Tilahun S. (2022). Changes in metabolites and antioxidant activities of green ‘Hayward’ and gold ‘Haegeum’ kiwifruits during ripening with ethylene treatment. Food Chem..

[110] McGhie T.K. (2013). Secondary metabolite components of kiwifruit. Adv. Food Nutr. Res..

[111] Xiong Y., He J., Li M., Du K., Lang H., Gao P., Xie Y. (2023). Integrative Analysis of Metabolome and Transcriptome Reveals the Mechanism of Color Formation in Yellow-Fleshed Kiwifruit. Int. J. Mol. Sci..

[112] Mertten D., Baldwin S., Cheng C.H., McCallum J., Thomson S., Ashton D.T., McKenzie C.M., Lenhard M., Datson P.M. (2023). Implementation of different relationship estimate methodologies in breeding value prediction in kiwiberry (*Actinidia arguta*). Mol. Breed..

[113] Wu J. (2020). Cultivar, environment and integration of cultural practices will determine the future of the kiwifruit industry. Scr. Hortic..

[114] Padhan A., Kumar A., Pathirana R., Sharma D.P., Thakur D.S., Rana V.S., Kumar P., Chauhan A. (2024). Potential of wild, underutilized *Actinidia callosa* and *Actinidia strigosa* from Northeast India for kiwifruit breeding. Genet. Resour. Crop Evol..

[115] Boopathi N.M., Boopathi N.M. (2020). Marker-assisted selection (MAS). Genetic Mapping and Marker Assisted Selection: Basics, Practice and Benefits.

[116] Kumawat G., Kumawat C.K., Chandra K., Pandey S., Chand S., Mishra U.N., Lenka D., Sharma R. (2020). Insights into marker assisted selection and its applications in plant breeding. Plant Breeding-Current and Future Views.

[117] Lei Y., Jing Z., Li L. Selection and evaluation of a new kiwifruit rootstock hybrid for bacterial canker resistance. Proceedings of the VIII International Symposium on Kiwifruit.

[118] Migicovsky Z., Myles S. (2017). Exploiting wild relatives for genomics-assisted breeding of perennial crops. Front. Plant Sci..

[119] Liu C., Zhang Q., Yao X., Zhong C., Yan C., Huang H. (2016). Characterization of genome-wide simple sequence repeats and application in interspecific genetic map integration in kiwifruit. Tree Genet. Genomes.

[120] Cheng C.-H., Datson P.M., Hanley Z. (2016). Genome-based breeding. Kiwifruit Genome.

[121] Wang Y., Liu Y. (2024). Recent advances of kwifruit genome and genetic transformation. Mol. Hortic..

[122] Alemu A., Åstrand J., Montesinos-Lopez O.A., y Sanchez J.I., Fernandez-Gonzalez J., Tadesse W., Vetukuri R.R., Carlsson A.S., Ceplitis A., Crossa J. (2024). Genomic selection in plant breeding: Key factors shaping two decades of progress. Mol. Plant.

[123] Varkonyi-Gasic E., Wang T., Voogd C., Jeon S., Drummond R.S., Gleave A.P., Allan A.C. (2019). Mutagenesis of kiwifruit CENTRORADIALIS-like genes transforms a climbing woody perennial with long juvenility and axillary flowering into a compact plant with rapid terminal flowering. Plant Biotechnol. J..

[124] De Mori G., Zaina G., Franco-Orozco B., Testolin R., De Paoli E., Cipriani G. (2020). Targeted mutagenesis of the female-suppressor SyGI gene in tetraploid kiwifruit by CRISPR/CAS9. Plants.

[125] Mohamed E., Adham N.E.S., Mohd Esa N.A.F., Abd Aziz M.H. (2022). Mutation in plant: Key of successful agriculture industry. Chemical Process and Sustainability In Agricultural Biotechnology.

[126] Lv H., Zhou Y., Tian H., Fei Z., Li D., Zhong C. (2024). New insights into colchicine-mediated tetraploidy in *Actinidia chinensis* ‘Donghong’. Hortic. J..

[127] Herath D., Voogd C., Mayo-Smith M., Yang B., Allan A.C., Putterill J., Varkonyi-Gasic E. (2022). CRISPR-Cas9-mediated mutagenesis of kiwifruit BFT genes results in an evergrowing but not early flowering phenotype. Plant Biotechnol. J..

[128] Zhong C., Wang S., Jiang Z., Huang H. (2012). ‘Jinyan’, an interspecific hybrid kiwifruit with brilliant yellow flesh and good storage quality. HortScience.

[129] Hirsch A., Testolin R., Brown S., Chat J., Fortune D., Bureau J., De Nay D. (2001). Embryo rescue from interspecific crosses in the genus *Actinidia* (kiwifruit). Plant Cell Rep..

[130] Michelotti V., Urbinati G., Gentile A., Lucioli S., Caboni E., Tacconi G. Preliminary results on the development of a genome editing protocol in Actinidia chinensis var. chinensis as Psa resistance approach. Proceedings of the X International Symposium on Kiwifruit.

[131] Salonia F., Ciacciulli A., Pappalardo H.D., Poles L., Pindo M., Larger S., Caruso P., Caruso M., Licciardello C. (2022). A dual sgRNA-directed CRISPR/Cas9 construct for editing the fruit-specific β-cyclase 2 gene in pigmented citrus fruits. Front. Plant Sci..

[132] Yasmeen E., Wang J., Riaz M., Zhang L., Zuo K. (2023). Designing artificial synthetic promoters for accurate, smart, and versatile gene expression in plants. Plant Commun..

[133] Rao G.S., Jiang W., Mahfouz M. (2021). Synthetic directed evolution in plants: Unlocking trait engineering and improvement. Synth. Biol..

[134] Butt H., Zaidi S.S.-e.-A., Hassan N., Mahfouz M. (2020). CRISPR-based directed evolution for crop improvement. Trends Biotechnol..

[135] Wang Y., Xue P., Cao M., Yu T., Lane S.T., Zhao H. (2021). Directed evolution: Methodologies and applications. Chem. Rev..

[136] Wan D.-Y., Guo Y., Cheng Y., Hu Y., Xiao S., Wang Y., Wen Y.-Q. (2020). CRISPR/Cas9-mediated mutagenesis of VvMLO3 results in enhanced resistance to powdery mildew in grapevine (*Vitis vinifera*). Hortic. Res..

[137] Malnoy M., Viola R., Jung M.-H., Koo O.-J., Kim S., Kim J.-S., Velasco R., Nagamangala Kanchiswamy C. (2016). DNA-free genetically edited grapevine and apple protoplast using CRISPR/Cas9 ribonucleoproteins. Front. Plant Sci..

[138] Xu X., Yuan Y., Feng B., Deng W. (2020). CRISPR/Cas9-mediated gene-editing technology in fruit quality improvement. Food Qual. Saf..

[139] Li C., Zhang R., Meng X., Chen S., Zong Y., Lu C., Qiu J.-L., Chen Y.-H., Li J., Gao C. (2020). Targeted, random mutagenesis of plant genes with dual cytosine and adenine base editors. Nat. Biotechnol..

[140] Nell H. (2007). Genetic Manipulation of Sucrose-Storing Tissue to Produce Alternative Products.

[141] Garst A.D., Bassalo M.C., Pines G., Lynch S.A., Halweg-Edwards A.L., Liu R., Liang L., Wang Z., Zeitoun R., Alexander W.G. (2017). Genome-wide mapping of mutations at single-nucleotide resolution for protein, metabolic and genome engineering. Nat. Biotechnol..

[142] Zhu H., Li C., Gao C. (2020). Applications of CRISPR–Cas in agriculture and plant biotechnology. Nat. Rev. Mol. Cell Biol..

[143] Li Y.-F., Jiang W., Liu C., Fu Y., Wang Z., Wang M., Chen C., Guo L., Zhuang Q.-G., Liu Z.-B. (2021). Comparison of fruit morphology and nutrition metabolism in different cultivars of kiwifruit across developmental stages. PeerJ.

[144] Lorestani A.N., Tabatabaeefar A. (2006). Modelling the mass of kiwi fruit by geometrical attributes. Int. Agrophys..

[145] Patterson K., Snelgar W., Richardson A., McPherson H. Flower quality and fruit size of Hayward kiwifruit. Proceedings of the IV International Symposium on Kiwifruit.

[146] Snelgar W., Manson P., Hopkirk G. (1991). Effect of overhead shading on fruit size and yield potential of kiwifruit (*Actinidia deliciosa*). J. Hortic. Sci..

[147] Jaeger S.R., Harker R., Triggs C.M., Gunson A., Campbell R.L., Jackman R., Requejo-Jackman C. (2011). Determining consumer purchase intentions: The importance of dry matter, size, and price of kiwifruit. J. Food Sci..

[148] Seal A. The plant breeding challenges to making kiwifruit a worldwide mainstream fresh fruit. Proceedings of the V International Symposium on Kiwifruit.

[149] Burdon J.N. (2018). Kiwifruit biology: The commercial implications of fruit maturation. Hortic. Rev..

[150] Montefiori M., McGhie T.K., Costa G., Ferguson A.R. (2005). Pigments in the fruit of red-fleshed kiwifruit (*Actinidia chinensis* and *Actinidia deliciosa*). J. Agric. Food Chem..

[151] Ma T., Sun X., Zhao J., You Y., Lei Y., Gao G., Zhan J. (2017). Nutrient compositions and antioxidant capacity of kiwifruit (*Actinidia*) and their relationship with flesh color and commercial value. Food Chem..

[152] Xia H., Wang X., Zhou Y., Su W., Jiang L., Deng H., Li M., Zhuang Q., Xie Y., Liang D. (2021). Biochemical and molecular factors governing flesh-color development in two yellow-fleshed kiwifruit cultivars. Sci. Hortic..

[153] Yan H., Chen H., Zhao J., Yao T., Ding X. (2023). Postharvest H_2_O_2_ treatment affects flavor quality, texture quality and ROS metabolism of ‘Hongshi’ kiwifruit fruit kept at ambient conditions. Food Chem..

[154] Cozzolino R., De Giulio B., Petriccione M., Martignetti A., Malorni L., Zampella L., Laurino C., Pellicano M. (2020). Comparative analysis of volatile metabolites, quality and sensory attributes of *Actinidia chinensis* fruit. Food Chem..

[155] Vanneste J. (2012). *Pseudomonas syringae* pv. actinidiae (Psa): A threat to the New Zealand and global kiwifruit industry. N. Z. J. Crop Hortic. Sci..

[156] Liu W., Zhao C., Liu L., Huang D., Ma C., Li R., Huang L. (2022). Genome-wide identification of the TGA gene family in kiwifruit (*Actinidia chinensis* spp.) and revealing its roles in response to *Pseudomonas syringae* pv. actinidiae (Psa) infection. Int. J. Biol. Macromol..

[157] Okada S., Kato-Noguchi H. (2021). Involvement of kiwifruit root autotoxicity in its replant problem. Plant Root.

[158] C Hunter D., Greenwood J., Zhang J., A Skinner M. (2011). Antioxidant and ‘natural protective’ properties of kiwifruit. Curr. Top. Med. Chem..

[159] Xu X.-B. (2003). Researches and utilizations of germplasm resource of kiwifruit in China. Chin. Bull. Bot..

[160] Yin X.-R., Allan A.C., Xu Q., Burdon J., Dejnoprat S., Chen K.-S., Ferguson I.B. (2012). Differential expression of kiwifruit ERF genes in response to postharvest abiotic stress. Postharvest Biol. Technol..

[161] Yuan X., Liang D., Wang X., Xia H. Kiwifruit Seedlings ‘Watt’ and ‘Hayward’ Physiological Response to Salt Stress. Proceedings of the 2018 3rd International Conference on Advances in Materials, Mechatronics and Civil Engineering (ICAMMCE 2018).

[162] Jing Z., Liu Z. (2018). Genome-wide identification of WRKY transcription factors in kiwifruit (*Actinidia* spp.) and analysis of WRKY expression in responses to biotic and abiotic stresses. Genes Genom..

[163] Zhao X., Xia H., Wang J., Lv X., Liang D. Effects of Exogenous Melatonin on Antioxidant Activity of Kiwifruit Leaves in Response to Drought Stress. Proceedings of the 2017 3rd International Forum on Energy, Environment Science and Materials (IFEESM 2017).

[164] Krishnappa G., Savadi S., Tyagi B.S., Singh S.K., Mamrutha H.M., Kumar S., Mishra C.N., Khan H., Gangadhara K., Uday G. (2021). Integrated genomic selection for rapid improvement of crops. Genomics.

[165] Lin M., Gao Z., Wang X., Huo H., Mao J., Gong X., Chen L., Ma S., Cao Y. (2024). Eco-friendly managements and molecular mechanisms for improving postharvest quality and extending shelf life of kiwifruit: A review. Int. J. Biol. Macromol..

[166] Lufu R., Ambaw A., Opara U.L. (2020). Water loss of fresh fruit: Influencing pre-harvest, harvest and postharvest factors. Sci. Hortic..

[167] Lara I., Belge B., Goulao L.F. (2014). The fruit cuticle as a modulator of postharvest quality. Postharvest Biol. Technol..

[168] Winkler A., Athoo T., Knoche M. (2022). Russeting of fruits: Etiology and management. Horticulturae.

[169] Paul V., Pandey R. (2014). Role of internal atmosphere on fruit ripening and storability—A review. J. Food Sci. Technol..

